# Synaptic spinules are reliable indicators of excitatory presynaptic bouton size and strength and are ubiquitous components of excitatory synapses in CA1 hippocampus

**DOI:** 10.3389/fnsyn.2022.968404

**Published:** 2022-08-11

**Authors:** Ashley Gore, Amaliya Yurina, Anastasia Yukevich-Mussomeli, Marc Nahmani

**Affiliations:** Division of Sciences and Mathematics, School of Interdisciplinary Arts and Sciences, University of Washington Tacoma, Tacoma, WA, United States

**Keywords:** electron microscopy, synaptic plasticity, presynaptic morphology, synapse structure, axo-axonic, transendocytosis, clathrin endocytosis

## Abstract

Synaptic spinules are thin, finger-like projections from one neuron that become embedded within the presynaptic or postsynaptic compartments of another neuron. While spinules are conserved features of synapses across the animal kingdom, their specific function(s) remain unknown. Recent focused ion beam scanning electron microscopy (FIB-SEM) image volume analyses have demonstrated that spinules are embedded within ∼25% of excitatory boutons in primary visual cortex, yet the diversity of spinule sizes, origins, and ultrastructural relationships to their boutons remained unclear. To begin to uncover the function of synaptic spinules, we sought to determine the abundance, origins, and 3D ultrastructure of spinules within excitatory presynaptic spinule-bearing boutons (SBBs) in mammalian CA1 hippocampus and compare them with presynaptic boutons bereft of spinules (non-SBBs). Accordingly, we performed a comprehensive 3D analysis of every excitatory presynaptic bouton, their embedded spinules, and postsynaptic densities, within a 5 nm isotropic FIB-SEM image volume from CA1 hippocampus of an adult male rat. Surprisingly, we found that ∼74% of excitatory presynaptic boutons in this volume contained at least one spinule, suggesting they are fundamental components of excitatory synapses in CA1. In addition, we found that SBBs are 2.5-times larger and have 60% larger postsynaptic densities (PSDs) than non-SBBs. Moreover, synaptic spinules within SBBs are clearly differentiated into two groups: small clathrin-coated spinules, and 29-times larger spinules without clathrin. Together, these findings suggest that the presence of a spinule is a marker for stronger and more stable presynaptic boutons in CA1, and that synaptic spinules serve at least two separable and distinct functions.

## Introduction

Synaptic spinules are thin, finger-like invaginating projections from one neuron that become wholly enveloped by the presynaptic or postsynaptic compartments of another neuron (Tarrant and Routtenberg, 1977; Geinisman et al., 1994; Spacek and Harris, 2004). These enveloped protrusions are evolutionarily conserved features across animal phyla, from invertebrate cnidarians (Westfall, 1970; Case et al., 1972) to humans (Zhang and Houser, 1999; Dall’Oglio et al., 2015). Indeed, recent data suggests they are ubiquitous components of mammalian excitatory cortical synapses (Campbell et al., 2020). Yet, synaptic spinule function(s) remain obscure due to a lack of investigations into their prevalence, morphological characteristics, and impact on synaptic function.

A majority of what we know about synaptic spinules comes from data on the protrusions emanating from the dendritic spines of pyramidal cells in the CA1 hippocampus and dentate gyrus of adult rat (Westrum and Blackstad, 1962; Tarrant and Routtenberg, 1977; Geinisman et al., 1994; Spacek and Harris, 2004). Most spinules in these regions seem to project from spines versus other neurites, and nearly all of these spinules are preferentially enveloped by presynaptic boutons (Geinisman et al., 1987; Spacek and Harris, 2004). Furthermore, some of the presynaptic bouton membranes surrounding these spinules are coated in clathrin (Tarrant and Routtenberg, 1977; Sorra et al., 1998; Spacek and Harris, 2004), suggesting an underexplored form of neuronal communication. In addition, the prevalence of spines projecting spinules increases following the induction of long-term potentiation (Schuster et al., 1990; Geinisman et al., 1994; Toni et al., 1999), indicating spinules may participate in activity-dependent information transfer and/or circuit remodeling.

However, investigations into spinules within CA1 hippocampus have focused on the spines projecting spinules rather than the prevalence and quantitative morphology of their presynaptic spinule-bearing bouton (SBB) partners. As such, basic questions regarding synaptic spinule structure and function in CA1 remain unexplored, such as: (1) where do spinules enveloped by SBBs originate? (2) what are the morphological features of spinules within SBBs? and (3) how do SBBs and the synapses they make differ from boutons without spinules (non-SBBs)? Here, we investigated these questions through a comprehensive 3D analysis of a 5 nm isotropic focused ion beam scanning electron microscopy (FIB-SEM) open data image volume from the CA1 hippocampus of an adult rat. By analyzing every excitatory presynaptic bouton, postsynaptic density (PSD), and spinule within this volume, we found that SBBs and non-SBBs are quantitatively distinct populations, and that spinules within SBBs come in two discrete “flavors” – small spinules with clathrin-coated tips, and large spinules without clathrin. Taken together, these data suggest that synaptic spinules serve at least two separable and distinct functions, and that the presence of a spinule is a reliable indicator of presynaptic bouton strength and stability within CA1 hippocampus.

## Materials and methods

### Material

All analyses were performed on a freely available 10.2 × 7.7 × 5.3 μm FIB-SEM image volume (5 nm isotropic voxel resolution) from the *stratum radiatum* of CA1 hippocampus of an adult male rat, originally acquired by Dr. Graham Knott. The protocol that was used for sample fixation, embedding, FIB-SEM preparation, and imaging of this tissue have previously been published (Knott et al., 2011). The full image volume analyzed here is available for download at: https://www.epfl.ch/labs/cvlab/data/data-em/.

### 3D analyses

**FIGURE 1:**
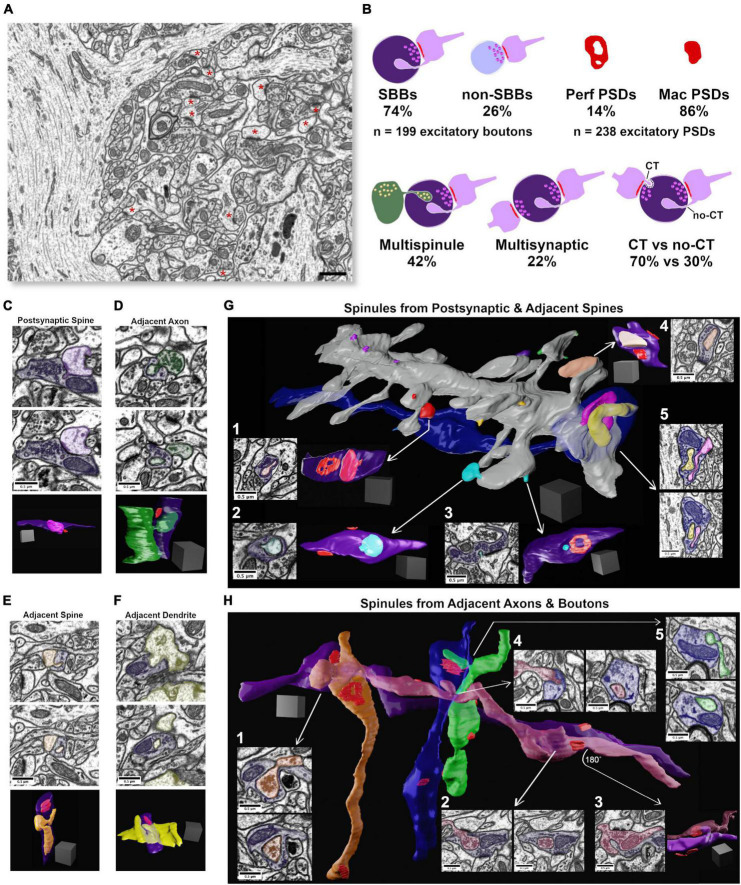
Synaptic spinules are fundamental components of excitatory synapses in CA1. **(A)** Single image from the 5 nm isotropic FIB-SEM image volume of CA1 hippocampus. Excitatory asymmetric postsynaptic densities (PSDs) are indicated by a red asterisk. Note the large apical pyramidal cell dendrites that flank the left and right sides of the image that reduce excitatory synapse density throughout this image volume. **(B)** Cartoon showing quantification of the percentage of excitatory presynaptic spinule-bearing boutons (SBBs), presynaptic boutons without spinules (non-SBBs), perforated postsynaptic densities (Perf PSDs), macular PSDs, SBBs enveloping multiple spinules, SBBs with multiple synapses, and spinules with clathrin-coated tips (CT) versus those without clathrin-coats (no-CT). **(C)** Representative example of an SBB (purple) with CT spinules from its postsynaptic spine (PSs; pink) partner. Upper and middle panels are adjacent FIB-SEM images showing the invagination and envelopment of one of three PSs spinules into this SBB. Bottom panel shows the 3D reconstruction of this PSs projecting three spinules into the SBB. Note that this SBB forms a second synapse (PSD in red) with a second spine (not shown). **(D)** Upper and middle panels show adjacent FIB-SEM images of an adjacent bouton (AdjAx, green) projecting a no-CT spinule into an SBB (purple) that has a synapse with a postsynaptic spine (uncolored PSD, top left of SBB). Bottom panel shows the 3D reconstruction of this AdjAx with its large no-CT spinule enveloped by the SBB, and the SBB’s PSD (red) with the postsynaptic spine (not shown). Note the second small CT AdjAx spinule projecting into the very bottom of this SBB. **(E)** Upper and middle panels show two FIB-SEM images of an adjacent spine (no PSD with this SBB; AdjS; orange) projecting (top panel) a large no-CT spinule into an SBB that becomes fully enveloped (lower panel). Bottom panel shows the 3D reconstruction of this AdjS, SBB, and SBB’s PSD (red). Note the spinule’s small (∼30 nm) invagination site (top and bottom panels) that expands into a large, enveloped structure that extends through the majority of this SBB’s length (middle and bottom panels). **(F)** Upper and middle panels show two successive FIB-SEM images of an adjacent dendrite (AdjD, yellow) projecting a no-CT spinule into an SBB (purple), with the spinule fully enveloped by the SBB (middle panel). Bottom panels show the 3D reconstruction of the surface of this dendrite and its non-synaptic spinule encapsulated by this SBB with a synapse (red) onto a dendritic spine from a separate dendrite (not shown). **(G)** 3D reconstruction of a segment of a dendrite and its spines, a majority of which project spinules into SBBs. All spinules from an individual spine are pseudocolored with the same color. Note that individual spines may project both CT and no-CT spinules (e.g., red and cyan spinules). (G_1_,G_2_) Single FIBSEM sections showing large no-CT spinules within SBBs (purple) emanating from distinct spines (arrows), and the 3D reconstruction of these SBBs (at right) displaying the enveloped spinule and PSDs (red). (G_3_) FIB-SEM image and 3D reconstruction showing a small CT spinule (cyan) enveloped by an SBB (purple), displaying an annulus-shaped perforated PSD (red). (G_4_) FIB-SEM image and 3D reconstruction showing an SBB enveloping a spine head spinule (peach) and displaying two macular PSDs (red). (G_5_) 3D reconstruction (arrow origin) and two successive FIB-SEM images showing an inhibitory presynaptic bouton (blue) with a synapse (symmetric PSD at left of bouton) onto a large pyramidal cell apical dendrite, and two enveloped spinules (pink and yellow) from nearby synaptic spines. **(H)** 3D reconstruction of four excitatory axons (orange, purple, magenta, and green), and one inhibitory axon (blue), projecting AdjAx spinules into each other, with visible PSDs (red). Arrows point to FIB-SEM images of the same SBB and AdjAx spinule relationships. (H_1_) Two successive FIB-SEM images showing an adjacent axon projecting a large no-CT spinule (orange) containing presynaptic vesicles into an SBB (purple). (H_2_) Two successive FIB-SEM images showing an adjacent presynaptic bouton (magenta) projecting a large no-CT spinule into an SBB (purple). Note that the individual synapses that both boutons have with postsynaptic spines are visible as dark PSDs at top and bottom of image at left. (H_3_) FIB-SEM image (left) and 3D reconstruction (right) showing the reciprocal relationship to panel (H_2_), with SBB from magenta axon enveloping an AdjAx spinule from the purple axon’s presynaptic bouton and PSDs shown in red. (H_4_) Two successive FIB-SEM images showing an AdjAx (magenta) with a spinule into an inhibitory presynaptic bouton (blue). (H_5_) Two successive FIB-SEM images showing another AdjAx (green) with a spinule into this same inhibitory presynaptic bouton (blue) with a synapse (symmetric PSD) onto a dendritic shaft (top FIB-SEM image and red PSD opposite green spinule in 3D reconstruction). Scale bars **(A)** 1 μm, **(C–H)** 0.5 μm; Scale cubes **(C–H)** 0.5 μm per side.

**FIGURE 2:**
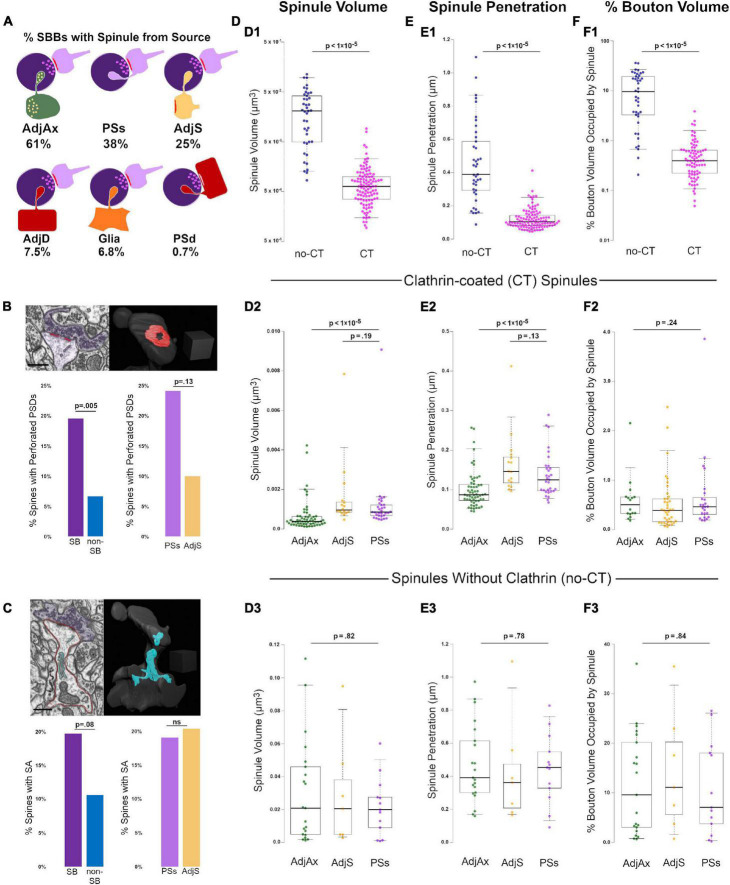
Clathrin-coated (CT) and uncoated spinules (no-CT) are distinct populations within CA1 SBBs. **(A)** Cartoon showing the percentage of SBBs containing at least one spinule from adjacent axons/boutons (AdjAx), postsynaptic spines (PSs), adjacent (no synapse with SBB) spines (AdjS), adjacent dendrites (AdjD), glia, and postsynaptic dendrites (PSd). Percentages reflect the ∼42% of SBBs with spinules from multiple sources. **(B)** Percentage of perforated PSDs in postsynaptic spines in this image volume. Upper left panel shows a FIB-SEM image of a postsynaptic spine (pink) with a perforated PSD (red) and its presynaptic SBB partner (purple). Note the CT spinule projecting just to the right of the PSD. Upper right panel shows 3D reconstruction of spine and PSD from FIB-SEM image; note this is the same spine as shown in Figures 1G_**2**_,G_**3**_. Lower left panel shows the percentages of spinule-bearing (SB) and non-spinule-bearing (non-SB) postsynaptic spines displaying perforated PSDs. Lower right panels show the percentages of spines projecting PSs spinules and AdjS spinules into SBBs that display perforated PSDs. **(C)** Percentage of spines with a spine apparatus in this image volume. Upper left shows a FIB-SEM image of the same postsynaptic spine (outlined in pink) as in panel **(B)**, displaying a spine apparatus (cyan), with a synapse onto an SBB (purple). Upper right panel shows the 3D reconstruction of this spine and its spine apparatus. Lower left panel shows the percentages of SB and non-SB spines displaying a spine apparatus, while lower right panels show the percentages of spines projecting PSs and AdjS spinules containing a spine apparatus. **(D)** Spinule volume comparisons between no-CT (blue) and CT spinules (pink) **(D_1_)**, between CT spinules of AdjAx (green), AdjS (yellow), and PSs origins (magenta) **(D_2_)**, and between no-CT spinules of AdjAx, AdjS, and PSs origins **(D_3_)**. For all boxplots, center lines show medians, box limits indicate the 25th and 75th percentiles, whiskers extend to the 5th and 95th percentiles, width of boxes is proportional to the square root of the sample size, and raw data points are plotted as filled circles. **(E)** Comparisons of spinule penetration distance into their SBBs between no-CT and CT spinules **(E_1_)**, between CT spinules of AdjAx, AdjS, and PSs origins **(E_2_)**, and between no-CT spinules of AdjAx, AdjS, and PSs origins **(E_3_)**. **(F)** Comparisons of the percentage of SBB volume occupied by no-CT and CT spinules **(F_1_)**, CT spinules of AdjAx, AdjS, and PSs origins **(F_2_)**, and no-CT spinules of AdjAx, AdjS, and PSs origins **(F_3_)**. Scale bars **(B,C)** 0.5 μm; Scale cubes **(B,C)** 0.5 μm per side.

**FIGURE 3:**
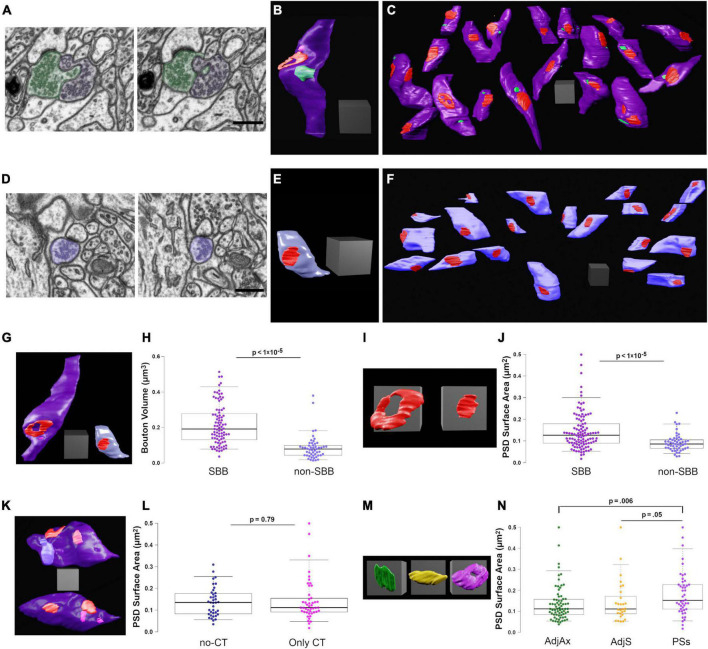
Spinule-bearing presynaptic boutons (SBBs) are larger and stronger than presynaptic boutons without spinules (non-SBBs). **(A)** Successive FIB-SEM images showing a spinule from an adjacent presynaptic bouton (AdjAx; green) with a spinule projecting into and enveloped by an SBB (purple). **(B)** 3D reconstruction of the same SBB (purple) and AdjAx spinule (green) as shown in panel **(A)**, displaying an annulus-shaped perforated PSD (red) and a CT spinule (pink) from a postsynaptic spine (not shown). **(C)** Field of 19 3D reconstructed SBBs (purple) in their approximate neuropil locations within the FIB-SEM image stack; some non-SBBs have been rotated to display their PSDs (red) and spinule invagination sites (green). **(D)** Successive FIB-SEM images showing a presynaptic non-SBB (light blue) with a synapse (dark PSD above bouton) onto a postsynaptic spine. Note that this postsynaptic spine is enveloped by an inhibitory presynaptic bouton (top of right FIB-SEM image) and has a long no-CT spinule into this inhibitory bouton (see yellow spinule in Figure 1G_**5**_). **(E)** 3D reconstruction of the same non-SBB (light blue) and PSD (red) as shown in panel **(D)**. **(F)** Field of 20 non-SBBs (light blue) in their approximate neuropil locations within the FIB-SEM image stack; some SBBs have been rotated to display their PSDs (red). **(G)** Same 3D reconstructions of SBB and non-SBB shown in panels **(B,E)**, shown side by side for comparison. **(H)** Box plots showing the presynaptic bouton volume for SBBs and non-SBBs (*n* = 87 and 51, respectively). For all boxplots, center lines show medians, box limits indicate the 25th and 75th percentiles, whiskers extend to the 5th and 95th percentiles, width of boxes is proportional to the square root of the sample size, and raw data points are plotted as filled circles. **(I)** Zoomed-in view of the 3D reconstructions in panel **(G)** showing PSDs from an SBB (left) and a non-SBB (right). **(J)** Boxplots of PSD surface area for SBBs and non-SBBs (*n* = 107 and 52, respectively). **(K)** 3D reconstructions of SBBs with no-CT (above, light blue) and CT (below, pink) spinules, displaying their PSDs (red). **(L)** Boxplots of PSD surface area for SBBs with only no-CT spinules and SBBs with only CT spinules. **(M)** 3D reconstructions of representative PSDs from SBBs containing AdjAx (green), adjacent spine (AdjS, yellow), and postsynaptic spine (PSs, magenta) spinules. **(N)** Boxplots of PSD surface area for SBBs containing spinules from AdjAx, AdjS, and PSs origins. Scale bars **(A,D)** 0.5 μm; All scale cubes = 0.5 μm per side.

Our detailed FIB-SEM analysis protocols have previously been published (Campbell et al., 2020; McCoy and Nahmani, 2021). Briefly, we used ImageJ (i.e., FIJI) to scroll through the image volume at 5 nm z-resolution in order to locate every excitatory synapse and spinule within excitatory boutons in the volume (Schindelin et al., 2012). All 3D analyses were performed and proofread by at least two reviewers to ensure accuracy of traces, the presence/absence of a spinule, and correct spinule origin assignment. Putative excitatory synapses were only included in our analyses if they had (1) parallel alignment of presynaptic and postsynaptic membranes at the active zone, (2) ≥3 presynaptic vesicles, (3) a prominent asymmetric postsynaptic density opposite these presynaptic vesicles, and (4) the presynaptic bouton was fully contained (i.e., not cut off) in the image volume. Moreover, presynaptic boutons whose volumes were truncated by the image volume were not included in any of our analyses. Presynaptic bouton volume was delineated based on the clustering of presynaptic vesicles: across its depth, a bouton was deemed to “end” when its vesicle cluster decreased to ≤2 vesicles, and across its width a bouton was deemed to “end” at the point where any vesicle was ≥2.5-vesicle widths away from its nearest neighbor. We analyzed each synapse for the identity of the postsynaptic neurite, the presence/absence of a spinule, the presence/absence of a perforated postsynaptic density, and the origin of the spinule (if present). We then assigned a regimented name to each presynaptic bouton based on these characteristics. Spinules were only included in our FIB-SEM analyses if they were observed invaginating into a presynaptic bouton and were then seen in at least one sequential image as a membrane-bound inclusion (i.e., unambiguous visualization of lipid bilayer of spinule surrounded by bouton’s lipid bilayer) within the presynaptic bouton. Spinule origins within FIB-SEM stacks were determined by tracking them within the image volume to their parent neurite or glial process. When it was not possible to identify the parent neurite/glia from which a spinule emerged (i.e., due to an object lacking one or more identifiable morphological criteria by the end of the image volume) we assigned this spinule to the “unknown” origin category. Approximately 7.5% of all SBBs (*n* = 11) contained a spinule from an “unknown” origin, though most of these SBBs (73%) contained at least one additional spinule from an identified source. PSDs were deemed “perforated” if they displayed one or more separations in the electron-dense region of the PSD, whereas macular PSDs were defined as PSDs bereft of these separations (Figures 1B, 2B, 3I). PSD separations needed to be seen in approximately the same x/y location in at least two consecutive serial FIBSEM images (i.e., 10 nm in the z dimension) to count as perforations. Every postsynaptic spine was evaluated for the presence of a spine apparatus, identified as tubules of smooth endoplasmic reticulum that needed to be present within both the spine neck and spine head (Figure 2C). Clathrin-coated spinules were detected by the presence of electron-dense “spikes” representing clathrin triskelions present on the cytoplasmic surface of invaginating bouton membranes (Figures 1C, 2B and Supplementary Figure 1). To analyze bouton, spinule, and PSD volumes and surface areas, we manually traced and reconstructed 87 SBBs with spinules from AdjAx, AdjS, and PSs parent origins, including their spinules and PSDs, and 51 non-SBBs with their PSDs, using Reconstruct software (Fiala, 2005). We three-dimensionally reconstructed every excitatory presynaptic non-SBB we encountered in this image volume. However, we excluded a number of SBBs from our 3D reconstructions due to a portion of a spinule and/or postsynaptic structure being cut off from the image volume (*n* = 27) or if an SBB contained a spinule of “unknown” origin (*n* = 11). In addition, we excluded SBBs containing spinules from AdjD (*n* = 11), Glia (*n* = 10), and PSd (*n* = 1) since their low prevalence prevented us from performing any meaningful statistical comparisons of their spinule and/or SBB sizes. 3D reconstruction surfaces were remeshed and transparency and lighting were adjusted using Blender open-source software.^1^

### Statistics

Statistical tests, means ± standard error of the mean (SEM), *p*-values, effect sizes, and statistical power for all comparisons are listed in Table 1. For all tests, α was set at 0.05, except where Bonferroni or Yates corrected *p*-values were used as appropriate and listed in Table 1.

**TABLE 1:** Means and descriptive statistics.

Comparison	Test	Mean ± SEM (n)	*P*-value^§^	Effect Size*	Power^†^
**Bouton volume** (μm^3^)					
SBB vs non-SBB	MW	SBB: 0.22 ± 0.01 (87); non-SBB: 0.09 ± 0.01 (51)	<0.00001	1.32	1.00
SBB only CT vs only no-CT	MW	CT: 0.20 ± 0.02 (47); no-CT: 0.20 ± 0.02 (30)	0.52		
SBB PSs vs AdjAx vs AdjS^א^	KW	PSs: 0.26 ± 0.02 (36); AdjAx: 0.22 ± 0.02 (58); AdjS: 0.26 ± 0.03 (23)	0.30		
**Bouton Surface Area** (μm^2^)					
SBB vs non-SBB	MW	SBB: 1.98 ± 0.09 (87); non-SBB: 0.88 ± 0.06 (51)	<0.00001	1.68	1.00
SBB CT vs no-CT	MW	CT: 1.79 ± 0.12 (47); no-CT: 2.01 ± 0.12 (30)	0.08		
SBB PSs vs AdjAx vs AdjS^א^	KW	PSs: 2.22 ± 0.15 (36); AdjAx: 1.99 ± 0.11 (58); AdjS: 2.24 ± 0.18 (23)	0.26		
**PSD Surface Area** (μm^2^)					
SBB vs non-SBB	MW	SBB: 0.14 ± 0.01 (107); non-SBB: 0.09 ± 0.01 (52)	<0.00001	0.71	0.98
SBB CT vs no-CT	MW	CT: 0.14 ± 0.01 (46); no-CT: 0.14 ± 0.01 (38)	0.79		
SBB PSs vs AdjAx vs AdjS^א^	KW	PSs: 0.18 ± 0.02 (46); AdjAx: 0.14 ± 0.01 (71); AdjS: 0.15 ± 0.02 (28)	0.02		
SBB PSs vs AdjAx	MW		0.006	0.42	0.57
SBB PSs vs AdjS	MW		0.048	0.29	0.20
SBB AdjAx vs AdjS	MW		0.90		
**% Spinule from Origin**					
% SBBs: PSs vs AdjAx vs AdjS	χ^2^	PSs: 38.1% (52); AdjAx: 60.5% (89); AdjS: 24.5% (36)	<0.00001		
%SBBs: PSs vs AdjAx	χ^2^		0.0001	0.32	0.97
%SBBs: PSs vs AdjS	χ^2^		0.01	0.21	0.71
%SBBs AdjAx vs AdjS	χ^2^		<0.00001	0.52	1.00
**% Multi-Spinule SBB**					
PSs vs AdjAx vs AdjS	χ^2^	PSs: 21.1% (31); AdjAx: 35.4% (52); AdjS: 14.3% (21)	0.93		
**Spinule Volume** (μm^3^)					
CT vs no-CT	MW	CT: 0.0009 ± 0.0001 (41); no-CT: 0.03 ± 0.004 (106)	<0.00001	1.98	1.00
CT: PSs vs AdjAx vs AdjS	KW	PSs: 0.0012 ± 0.0003 (31); AdjAx: 0.0006 ± 0.0001 (59); AdjS: 0.0016 ± 0.0004 (16)	<0.00001		
CT: PSs vs AdjAx	MW		<0.00001	0.38	0.38
CT: PSs vs AdjS	MW		0.19		
CT: AdjAx vs AdjS	MW		<0.00001	0.63	0.58
no-CT: PSs vs AdjAx vs AdjS	KW	PSs: 0.02 ± 0.005 (13); AdjAx: 0.03 ± 0.007 (21); AdjS: 0.03 ± 0.01 (7)	0.82		
**Spinule penetration** (μm)					
CT vs no-CT	MW	CT: 0.12 ± 0.01 (106); no-CT: 0.45 ± 0.04 (41)	<0.00001	1.90	1.00
CT: PSs vs AdjAx vs AdjS	KW	PSs: 0.14 ± 0.06 (31); AdjAx: 0.10 ± 0.01 (59); AdjS: 0.16 ± 0.02 (16)	<0.00001		
CT: PSs vs. AdjAx	MW		0.0003	0.72	0.88
CT: PSs vs. AdjS	MW		0.13		
CT: AdjAx vs AdjS	MW		<0.00001	0.90	0.87
no-CT: PSs vs AdjAx vs AdjS	KW	PSs: 0.44 ± 0.06 (13); AdjAx: 0.47 ± 0.05 (21); AdjS: 0.42 ± 0.12 (7)	0.78		
**Spinule: Bouton Volume**					
CT vs no-CT^∫^	MW	CT: 0.006 ± 0.0007 (79); no-CT: 0.12 ± 0.02 (41)	<0.00001	9.45	1.00
CT: PSs vs AdjAx vs AdjS^∫^	KW	PSs: 0.007 ± 0.002 (24); AdjAx: 0.005 ± 0.001 (39); AdjS: 0.006 ± 0.001 (16)	0.24		
no-CT: PSs vs AdjAx vs AdjS^∫^	KW	PSs: 0.11 ± 0.03 (13); AdjAx: 0.12 ± 0.02 (21); AdjS: 0.14 ± 0.05 (7)	0.84		
**% Spinules with Coated Tip**					
PSs vs AdjAx vs AdjS	χ^2^	PSs: 70.7% (99); AdjAx: 68.7%(194); AdjS: 72.5% (69)	0.98		
**% Spine Apparatus**					
SB vs. non-SB Spines	χ^2^	SB Spines: 19.8% (91); non-SB Spines: 10.7% (150)	0.05 (0.08)¥	0.127	0.50
PSs vs AdjS	χ^2^	PSs: 19.2% (52); AdjS: 20.5% (39)	0.91¥		
**% Perf PSDs**					
SB vs. non-SB Spines	χ^2^	SB Spines: 19.6% (92); non-SB Spines: 6.7% (150)	0.005¥	0.182	0.81
PSs vs AdjS	χ^2^	PSs: 24.1% (58); AdjS: 10.0% (40)	0.13¥		

^§^χ set at 0.05, Bonferroni corrected value = 0.025 and 0.017 for two and three group comparisons, respectively. **^†^***Post-hoc* achieved power at α = 0.05, two-tailed; *Hedges’ g for MW, *w* for χ^2^; MW = Mann–Whitney U test; KW = Kruskal-Wallis test; χ^2^ for Chi-Square test. ¥ Yates corrected *p*-value; ^א^Some SBBs are counted multiple times in this comparison because they contain spinules from multiple origins. ^∫^If multiple spinules of the same type (e.g., CT) were present within the same bouton, their volumes were summed for this comparison. Note that since ∼22% of SBBs and 2% of non-SBBs are multisynaptic, numbers of PSDs are greater than numbers of boutons. SBB, Spinule-Bearing Bouton; CT, Clathrin-coated spinule; SB, spinule-bearing; PSs, postsynaptic spine; AdjAx, adjacent axon; AdjS, adjacent spine; Perf, perforated.

## Results

### Synaptic spinules are fundamental components of excitatory synapses in CA1

To investigate the origins, ultrastructural diversity, and prevalence of spinules with hippocampal excitatory SBBs, we took advantage of a freely available 10.2 × 7.7 × 5.3 μm FIB-SEM image volume with 5 nm isotropic voxel resolution, from *stratum radiatum* of CA1 hippocampus of an adult male rat (Figure 1A). To determine the prevalence of spinules within the excitatory boutons in this data set, we performed a 3D analysis to locate every excitatory presynaptic bouton within this image volume that contained a fully enveloped spinule (see Methods). Surprisingly, we found that of the total excitatory presynaptic boutons (*n* = 199) in this CA1 image volume, a large majority (∼74%) contained at least one spinule, while a minority lacked spinules (∼26%; Figure 1B). By tracing each spinule back to the neurite it emanated from within the 3D image volume, we determined that spinules embedded within these SBBs originated from a diverse array of sources, including: spinules projected from postsynaptic spines (PSs, Figure 1C), adjacent axons/boutons (AdjAx, Figure 1D), adjacent (i.e., no synapse with SBB) spines (AdjS, Figure 1E), adjacent dendritic shafts (AdjD, Figure 1F), postsynaptic dendrites (PSd), glia, and a small percentage of indeterminate origin (7.5%; see the section “Methods”). SBBs predominantly contained spinules from just three sources, AdjAx (61%), PSs (38%), and AdjS (25%), with ≤7.5% of SBBs containing a spinule from any other single source (Figure 2A and Table 1). Note that these percentages reflect the ∼42% of SBBs that contained more than one spinule (mean = 1.7, range = 1 – 6, spinules per SBB). Interestingly, AdjAx spinules often contained presynaptic vesicles and/or a smooth endoplasmic reticulum within their lumens, a possible route for communication between boutons (Figures 1D,H_**1**_,H_**2**_). Furthermore, while our analyses were primarily focused on excitatory synapses, we observed inhibitory presynaptic boutons with enveloped PSs and AdjAx spinules from excitatory neurons (Figures 1G_**5**_,H_**4**_,H_**5**_ and Supplementary Video 1), demonstrating the potential for novel forms of excitatory to inhibitory neuron communication. These data suggest that spinules are prominent components of excitatory synapses in CA1 hippocampus, and that most spinules within SBBs come from dendritic spines, presynaptic boutons, and axons.

During our analysis, it became apparent that spinules within SBBs often displayed clathrin-coats along the leading edge of the invaginating presynaptic membrane (Figures 1B,C,G_**3**_), as previously observed in rat CA1 (Spacek and Harris, 2004). These clathrin-tipped (CT) spinules appeared relatively small, versus spinules without clathrin (no-CT); CT spinules emanated from every identified source of spinules except postsynaptic dendritic shafts. There were no detectable differences in the percentages of CT spinules from the three main spinule sources (*p* = 0.98, χ^2^ = 0.04; 68.7%, 72.5%, 70.7%, for AdjAx, PSs, and AdjS spinules, respectively). We quantified the percentage of SBBs (*n* = 147) containing at least one CT spinule versus those only containing no-CT spinules and found that a majority of SBBs contained CT spinules (70.1 vs. 29.9%, CT vs. no-CT, respectively, *n* = 302 spinules from all sources; Figure 1B). In addition, we found that while most presynaptic boutons had postsynaptic partners whose PSDs (*n* = 238 total PSDs) displayed a macular morphology (∼86%), some of the PSDs associated with these excitatory presynaptic boutons displayed perforations (∼14%, Figure 1B), similar to a previous 3D analysis of excitatory PSDs in adult rat CA1 (8 – 12%; (Sorra et al., 1998).

We next sought to determine if there were quantitative differences between spinules from different origins, or between spinules with and without a clathrin coat, that might hint at their function. Accordingly, we restricted the rest of our analyses to SBBs containing spinules from the three major neurite origins (AdjAx, PSs, and AdjS) to achieve reasonable statistical power across our comparisons (see the section “Methods” and Table 1). Furthermore, we limited our analysis to SBBs whose full spinule 3D volumes, and those of their postsynaptic partner(s), were fully contained within this image volume (*n* = 87 SBBs). Thus, while we analyzed 199 excitatory presynaptic boutons for the presence/origin of a spinule, we three-dimensionally reconstructed 87 SBBs and 51 non-SBBs (along with their 107 and 52 PSDs, respectively), and excluded 60 SBBs from further analysis (see the section “Methods”).

### Clathrin-coated and uncoated spinules are distinct populations

To determine whether spinules from different origins or those with clathrin coating are morphologically distinct, we three-dimensionally reconstructed 87 SBBs containing 147 AdjAx, PSs, and AdjS spinules. All statistical tests, sample sizes (n), *p*-values, effect size, and power for these and all further comparisons can be found in Table 1. Our analyses revealed that no-CT spinules were 29-times larger, projected 3.8-times farther into their SBB, and occupied 21-times more volume than CT spinules (*p* < 1 × 10^–5^; Figures 2D_**1**_–F_**1**_), indicating that CT and no-CT spinules are separate populations. We reasoned that if CT and no-CT spinules that emanate from different origins have distinct functions (e.g., large spinules that enhance synaptic stability vs. small spinules designed for transendocytosis), then they would likely display distinct morphologies. Thus, we compared CT spinules and no-CT spinules across origin categories (i.e., AdjAx, PSs, and AdjS). We found that CT spinules from AdjAx are ∼2-times smaller than CT spinules from PSs and AdjS (*p* < 1 × 10^–5^; Figures 2D_**2**_–F_**2**_), suggesting CT spinules from boutons and axons may have distinct functions from CT spinules originating from spines. However, AdjAx, PSs, and AdjS CT spinules occupied a similar percentage of their SBBs, indicating that AdjAx CT spinules may preferentially target SBBs that are smaller than those containing PSs and AdjS spinules. Although no-CT spinules were larger than their CT spinule counterparts, no-CT spinules from AdjAx, AdjS, and PSs origins were indistinguishable from each other in volume, bouton penetration, and the percent volume they occupied within their SBBs (*p* = 0.80; Figures 2D_**3**_–F_**3**_). Together, these results argue for two separable and distinct forms of spinules within the excitatory SBBs in CA1: small spinules participating in clathrin-mediated transendocytosis and large spinules devoid of clathrin that invaginate deep (∼450 nm) into their enveloping SBBs.

### Spinule-bearing spines are associated with perforated postsynaptic densities and spine apparatuses

Perforated PSDs are associated with increases in cortical activity (Calverley and Jones, 1990; Geinisman et al., 1993; Toni et al., 1999), and recent findings have suggested that spinule-bearing spines have higher rates of perforated PSDs (Campbell et al., 2020; Zaccard et al., 2020). Thus, we next sought to examine whether PSs and AdjS spinule-bearing spines (SB spines, *n* = 92) differed from spines without spinules (non-SB spines, *n* = 150). We found that while only 6.7% of non-SB spines had perforated PSDs, 19.6% of SB spines had perforated PSDs (*p* = 0.005). Interestingly, spines with PSs spinules showed a trend toward higher rates of perforated PSDs versus spines projecting AdjS spinules (24.1% vs. 10.0%, *p* = 0.13, *n* = 58 and 48, for PSs vs. AdjS, respectively; Figure 2B). However, given our relatively small sample size, this latter result awaits confirmation from a larger dataset (see Discussion). In sum, these findings intimate that spinules emanating from spines play a role in the dynamic partitioning of the PSD.

The presence of a spine apparatus is also associated with large spines that have recently experienced high levels of synaptic activity (Chirillo et al., 2019; Perez-Alvarez et al., 2020). Accordingly, we quantified the percentages of SB and non-SB spines containing a spine apparatus. We found that 19.8% of SB spines had a spine apparatus, while only 10.7% of non-SB spines had a spine apparatus (*p* = 0.08; *n* = 91 and 150, SB and non-SB spines, respectively, Figure 2C and Supplementary Video 3). PSs and AdjS projecting spines had similar percentages of spine apparatuses (19.2% vs. 20.5%, *p* = 0.91). Thus, SB spines showed a trend toward displaying an organelle associated with robust levels of synaptic activity at higher percentages than non-SB spines.

### Synaptic spinules are markers of stronger synapses

Our findings on spinule populations in CA1 highlighted the diversity of spinule morphologies, associated postsynaptic organelles, and their potential functions, prompting us to ask whether select presynaptic boutons (e.g., SBBs containing large no-CT spinules) might be larger than SBBs with small spinules or non-SBBs. Thus, we measured the volume and surface areas of SBBs containing AdjAx, AdjS, and PSs spinules (*n* = 87) and presynaptic non-SBBs (*n* = 51; Figures 3A–F). We found that SBBs had 2.5-times larger volumes and 2.3-times larger surface areas than their non-SBB counterparts (*p* < 1 × 10^–5^; Figures 3G,H). However, we did not detect an influence of spinule origin on SBB size (*p* = 0.30 and.26, for PSs vs. AdjAx vs. AdjS volume and surface area, respectively, Table 1). We next compared the PSD surface areas of SBBs versus non-SBBs as a measure of their respective synaptic strengths (Branco et al., 2010; Holderith et al., 2012; Araya et al., 2014; Meyer et al., 2014; Holler et al., 2021). Similar to SBB volume and surface area, we found that PSD surface areas at SBB synapses were 1.6-times larger than PSD surface areas at non-SBB synapses (*p* < 1 × 10^–5^, *n* = 107 and 52 for SBB and non-SBB PSDs, respectively; Figures 3I,J). Hence within rat CA1, the presence of a spinule within an excitatory presynaptic bouton is a marker for a larger and stronger synaptic contact.

Since we had determined that no-CT spinules were 29-times larger than CT spinules, we hypothesized that SBBs enveloping no-CT spinules might be larger than those containing CT spinules. Yet surprisingly, in comparing SBBs that only contained small CT spinules (*n* = 47) with those that only contained larger no-CT spinules (*n* = 30), there were no detectable differences in their volumes (*p* = 0.52; Table 1) or their PSD surface areas (*p* = 0.79; Figures 3K,L). However, we found a small but intriguing trend for no-CT containing SBBs possessing larger surface areas than SBBs with CT spinules (*p* = 0.08, 2.01 ± 0.12 vs. 1.79 ± 0.12, for no-CT and CT, respectively), intimating that large spinules might impact the morphological complexity of their SBBs. In addition, in comparing the PSD surface areas from SBBs containing spinules from AdjAx, AdjS, and PSs sources, we found that PSDs from SBBs with PSs spinules were 33% larger than PSDs from SBBs containing AdjAx spinules and showed a trend toward having larger PSDs than SBBs containing AdjS spinules (*p* = 0.006 and 0.05 for PSs vs. AdjAx and AdjS, respectively, Figures 3M,N). Together, these data suggest that spinule presence, regardless of size, is predictive of a distinct class of larger and stronger presynaptic boutons, and that SBBs with PSs spinules may possess the largest PSDs within the CA1 SBB population.

## Discussion

In this study, we sought to answer three questions: (1) Where do the spinules within excitatory SBBs in CA1 originate? (2) Do spinules within these SBBs display distinct morphologies? (3) How do SBBs and their PSDs differ from non-SBBs? Our 3D analyses of all the excitatory synapses within this CA1 FIB-SEM image volume revealed that spinules are ubiquitous within excitatory presynaptic in CA1, and that spinules within SBBs predominantly originate from three sources: adjacent excitatory axons/boutons, postsynaptic dendritic spines, and adjacent (non-synaptic) spines. In addition, we found that spinules come in two main categories: small clathrin-coated projections that are likely involved in transendocytosis (Spacek and Harris, 2004), and 29-times larger uncoated spinules that project deep into their host SBBs. Furthermore, we show that SBBs are 2.5-times larger and have 60% larger PSDs than their non-SBB counterparts within the same image volume. Together, these findings demonstrate that the presence of an encapsulated spinule is a robust predictor of excitatory presynaptic bouton size and strength, differentiating large and strong SBBs from smaller, weaker, and potentially less stable non-SBBs. Moreover, these data intimate that spinules are prominent components of CA1 excitatory synapses and serve at least two functions: small spinules involved in macromolecular communication through clathrin-mediated vesicular transendocytosis, and large spinules that significantly increase neuron-to-neuron extrasynaptic membrane interface to potentially enhance connection stability and/or allow for a unique form(s) of neuronal communication.

### Experimental limitations

These 3D analyses were conducted on one image volume from the CA1 hippocampus of an adult male rat. We chose this approach because it allowed us to manually analyze every excitatory bouton and synaptic spinule within an image volume of CA1 within reasonable time constraints. We concluded that a manual analysis approach in an image volume with high spatial resolution (i.e., <15 nm voxels) was best suited to our questions because spinule invagination sites into SBBs are often less than <40 nm wide (e.g., Figures 1C–E,H_**5**_). However, while we may have captured some of the variability inherent within this animal’s excitatory synapses in CA1, we could not capture inter-animal variability since these data were from a single animal. For example, this image volume will likely have a lower excitatory synapse density than some other volumes of CA1 due to the large apical pyramidal dendrites that flank the majority of its images (Figure 1A), at times reducing the area of synaptic neuropil by >50%. However, the averages and ranges for the excitatory bouton volumes and PSD surface areas we analyzed in this volume are consistent with previous 3D ultrastructural analyses of excitatory presynaptic boutons in rat CA1 (Shepherd and Harris, 1998; Grillo et al., 2018), indicating that the synaptic ultrastructure within our image volume is comparable with that of other analyses of the excitatory synapse population in rat CA1.

Our analyses revealed large differences in bouton volume and surface area between SBBs and non-SBBs, SBB and non-SBB PSDs, and CT vs. no-CT spinules (Table 1). Yet, as we expanded our analyses to include combinations of multiple variables/characteristics (e.g., perforated synapses from AdjS containing SBBs), our sample size was necessarily restricted, effectively decreasing our statistical power for two noted comparisons: the percentage of spine apparatuses within SB and non-SB spines, and the percentage of perforated PSDs from PSs and AdjS spines. Since the overall percentage of spines that contain a spine apparatus or perforated PSD in CA1 is small (7–20% in this study), it is difficult to achieve high levels of statistical power when comparing subgroups of spines (e.g., AdjS projecting spines) even when the computed difference between groups is large (e.g., twofold increase). For example, to achieve a statistical power of 0.8 for our spine apparatus comparisons, given the twofold difference between groups we observed (i.e., χ^2^ effect size = 1.3–1.4), would require a doubling of the total area used in this analysis. As such, these two comparisons (% spine apparatus between SB and non-SB spines, and % perforated PSDs between PSs and AdjS spines), while intriguing, await confirmation or refutation from future analyses of large volume high resolution (<15 nm voxel) FIB-SEM datasets.

### Spinule-bearing boutons and the potential for axo-axonic communication

In our previous 3D analysis of SBBs within a FIBSEM image volume of late adolescent ferret primary visual cortex, we found that the primary sources of spinules within SBBs were from AdjAx (35.2%), PSs (37.0%), AdjD (14.8%), and AdjS (7.4%) (Campbell et al., 2020). Thus, while SBBs in each brain area may preferentially envelop small percentages of spinules from specific sources based on distinct microcircuit functions, AdjAx and PSs spinules seem to be primary sources for spinules within SBBs across brain areas. Indeed, AdjAx spinules are the primary origin for spinules within SBBs across the postnatal development of ferret primary visual cortex (Campbell et al., 2020). Interestingly, it has been reported that only ∼12% of spinules emerge from axons and presynaptic boutons in adult rat CA1, with ∼86% projected from dendritic spines (Spacek and Harris, 2004). Here, we found that 61% of SBBs in adult CA1 contained spinules that originated from adjacent axons and other presynaptic boutons. This discrepancy in the percentage of spinules emerging from adjacent axons and those enveloped by SBBs could be due to our ability (with 5 nm isotropic voxel resolution) to detect spinules with invagination widths that were smaller than the section thickness (50 nm) used in the only other systematic investigation of spinules origins in CA1 (Spacek and Harris, 2004). Regardless, data from this study and a previous analysis, from a combined five FIB-SEM image volumes, demonstrate that AdjAx spinules are a primary source of spinules within excitatory SBBs in adult rat CA1 and developing ferret primary visual cortex (Campbell et al., 2020).

Indeed, these findings raise the possibility that axo-axonic communication may be more common than we currently understand. While there are examples of axo-axonic communication across brain regions (Cover and Mathur, 2021), axo-axonic synaptic contacts likely comprise a small minority of the total excitatory and inhibitory synapses across the brain (Somogyi et al., 1982; DeFelipe and Fariñas, 1992; Inan et al., 2013). Thus, if as we’ve shown 45% of CA1 excitatory synapses contain AdjAx spinules (i.e., 74% of presynaptic boutons are SBBs, and 61% of these contain AdjAx spinules), and there are ∼30,000 synapses per pyramidal neuron (Megías et al., 2001), each CA1 pyramidal neuron would have ∼13,500 presynaptic boutons that envelop and putatively communicate (see *Putative synaptic spinule functions* below) with other axons and boutons. Furthermore, our observations of presynaptic inhibitory SBBs enveloping excitatory axons and spines (Figures 1G_**5**_,H_**4**_,H_**5**_ and Supplementary Video 2), highlights the intriguing possibility that spinules may play a role in the rapid modulation of excitatory – inhibitory balance within CA1 microcircuits (Chevaleyre and Piskorowski, 2014).

### Synaptic spinules predict synapse strength

To our knowledge, only two other studies have examined the size of presynaptic boutons containing spinules, both presenting 2D evidence that SBBs are larger than adjacent non-SBBs (Erisir and Dreusicke, 2005; Campbell et al., 2020). Here, our 3D analyses revealed that SBB volumes and PSD surface areas are 2.5 and 1.6-times the size of non-SBBs, respectively, such that ∼50% of SBBs had volumes larger than 0.2 μm^3^ and PSD areas larger than 0.12 μm^2^, while only 4% of non-SBB volumes and 13% of their PSDs reached that size (Figures 3G–J). Accordingly, since presynaptic bouton volume and PSD surface area are directly related to synaptic strength and stability (Takumi et al., 1999; Murthy et al., 2001; Ostroff et al., 2002; Branco et al., 2010; Holderith et al., 2012; Araya et al., 2014; Meyer et al., 2014; Quinn et al., 2019; Holler et al., 2021), we argue that SBBs represent the strongest and most stable excitatory presynaptic boutons within rat CA1.

### Putative synaptic spinule functions

Surprisingly, we found that SBBs containing either small CT or large no-CT spinules were over 2 times larger than non-SBBs. That is, spinule size seems to be a poor predictor of SBB size, arguing against a simple stochastic relationship between spinule invagination and consequential SBB enlargement. Rather, this finding suggests two possibilities, either: (1) spinules aid in the stabilization and/or functional maturation of presynaptic boutons, or (2) spinules preferentially target and invaginate into already larger and stronger boutons. Regardless, the extreme differences in CT vs. no-CT spinule sizes and penetration strongly suggest they serve distinct functions. On average, large no-CT spinules project 450 nm into their SBBs (range: 90 nm – 1 μm) and occupy 12% of their SBBs’ volume. Thus, the process of no-CT spinule envelopment (i.e., spinule projection and SBB invagination) involves a substantial level of actin cytoskeleton rearrangement and lipid bilayer modulation that together represent a large energy drain on the spinule-projecting and spinule-receiving neurons (Bernstein and Bamburg, 2003; Engl and Attwell, 2015). This level of energy and anatomical investment suggests a long-term relationship, with no-CT spinules potentially acting as structural anchors to enhance the stability of these non-canonical neuronal connections. Indeed, *in vitro*, the largest spinules are also the most stable (Zaccard et al., 2020). Additionally, SBBs share 2.4-times more parallel membrane interface with their large no-CT spinules than they do with their own PSDs (0.36 (spinule) vs. 0.15 (PSD) μm^2^), making this interface a potential site for transmembrane communication (Wang et al., 2004). Alternatively, this large high-resistance membrane interface might allow for potent ephaptic communication between no-CT spinule projecting and receiving neurons, as large horizontal cell spinules do in vertebrate retina (Wagner and Djamgoz, 1993; Vroman et al., 2013). Finally, small CT spinules almost certainly transfer proteins and lipid bilayer between spinule and SBB, with the transendocytosis rate of these CT spinules increasing with high levels of neuronal activity (Sorra et al., 1998; Toni et al., 1999; Tao-Cheng et al., 2009).

## Data availability statement

The raw data supporting the conclusions of this article will be made available by the authors, without undue reservation.

## Author contributions

MN acquired the data, devised experiments, helped analyze data, and wrote the manuscript. AG, AY, and AY-M analyzed the data, prepared figures, and edited the manuscript. All authors contributed to the article and approved the submitted version.

## Footnotes

1
https://www.blender.org/

## Supplementary material

Supplementary Figure 1 Representative examples of clathrin-coated spinules. **(A_1_,A_2_)** Two consecutive FIB-SEM images showing a postsynaptic spine invaginating a clathrin coat-tipped (CT) spinule into an SBB. Red arrows point to the “spiked” looking clathrin triskelions surrounding the invaginating SBB membrane. **(A_3_)** Enlargement of the CT spinule shown in panel **(A_2_)**, red arrow points to clathrin coat. **(B_1_,B_2_)** Consecutive images showing an adjacent (non-synaptic) spine with a CT spinule projecting into an SBB. Red arrows point to clathrin coat. **(B_3_)** Enlargement of CT spinule shown in panel **(B_2_)**, with red arrow pointing to clathrin coat. **(C_1_,C_2_)** Two consecutive images of an adjacent axon projecting a CT spinule into an SBB. Red arrows point to clathrin coats. **(C_3_)** Enlargement of CT spinule shown in panel **(C_2_)** with red arrow pointing to clathrin coat. **(D_1_,D_2_)** Two consecutive images showing the lower end of a postsynaptic spine invaginating a CT spinule into an SBB. Red arrows point to clathrin coats. **(D_3_)** Enlargement of CT spinule shown in panel **(D_2_)**, showing clathrin coat (red arrow) surrounding CT spinule from postsynaptic spine. Scale bars **(A_2_,A_3_,B_2_,B_3_,C_2_,C_3_,D_2_)** = 0.5 μm; Scale bar **(D_3_)** = 0.25 μm.

Supplementary Video 1 Animation showing the pseudocolored CT and no-CT spinules present on the dendritic spines from Figure 1G.

Supplementary Video 2 Animation showing the adjacent axon and bouton spinules present within SBBs, as shown in Figure 1H.

Supplementary Video 3 FIB-SEM image volume animation showing the inhibitory bouton from Figure 1G_**5**_ (lower left of animation) receiving spinules from two spines (red arrows). The center of this FIB-SEM animation shows the large mushroom spine containing a spine apparatus shown in Figures 2B,C. Note the small clathrin-coated CT spinule it projects into its SBB partner (red arrow).
