# Supplementary figures and images for: Synaptic spinules are reliable indicators of excitatory presynaptic bouton size and strength and are ubiquitous components of excitatory synapses in CA1 hippocampus
Source: Front Synaptic Neurosci. 2022 Aug 11;14:968404. doi: 10.3389/fnsyn.2022.968404 (PMC9403541; doi:10.3389/fnsyn.2022.968404)

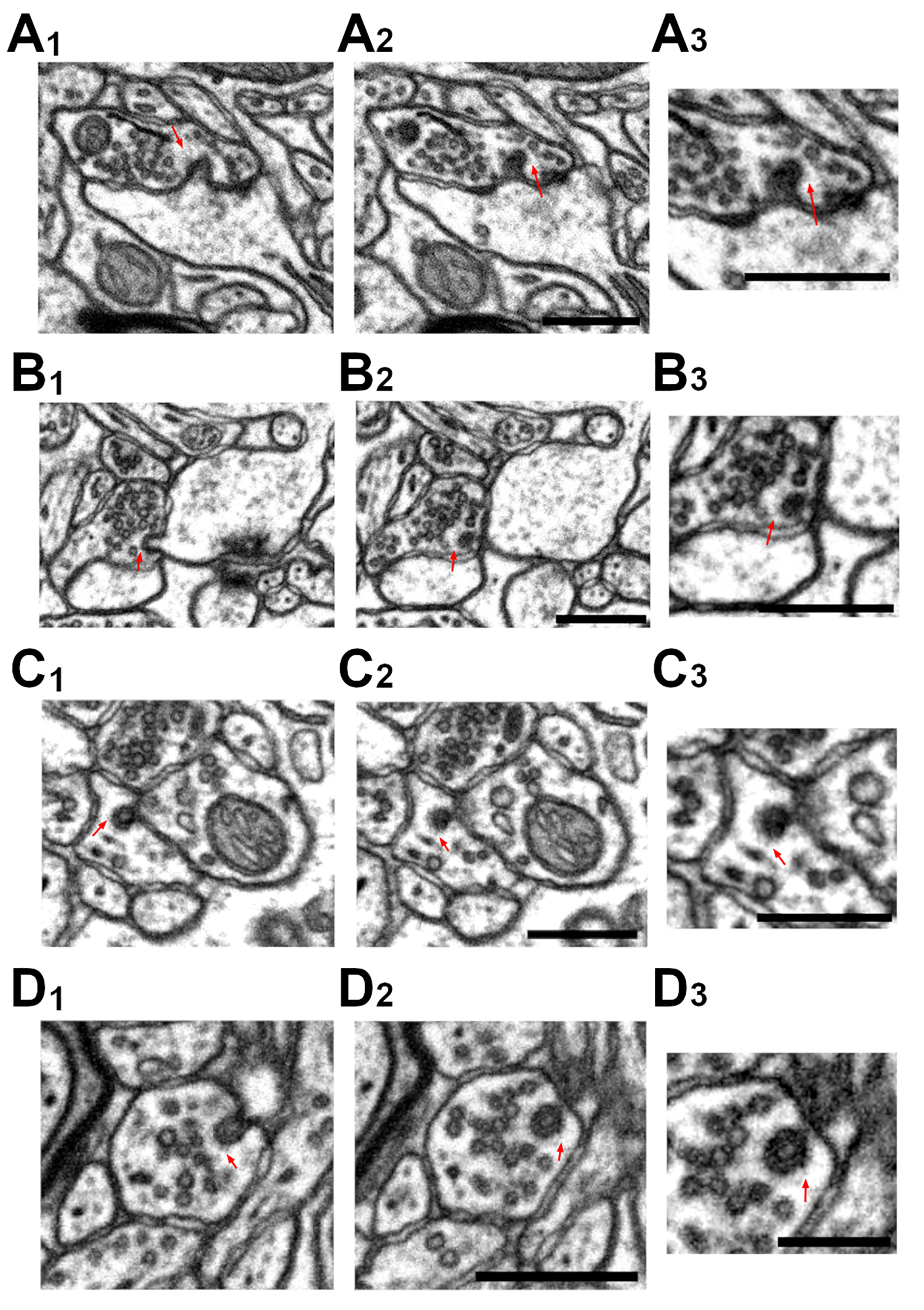

Supplement: Supplementary Figure 1 — Representative examples of clathrin-coated spinules. (A1,A2) Two consecutive FIB-SEM images showing a postsynaptic spine invaginating a clathrin coat-tipped (CT) spinule into an SBB. Red arrows point to the “spiked” looking clathrin triskelions surrounding the invaginating SBB membrane. (A3) Enlargement of the CT spinule shown in panel (A2), red arrow points to clathrin coat. (B1,B2) Consecutive images showing an adjacent (non-synaptic) spine with a CT spinule projecting into an SBB. Red arrows point to clathrin coat. (B3) Enlargement of CT spinule shown in panel (B2), with red arrow pointing to clathrin coat. (C1,C2) Two consecutive images of an adjacent axon projecting a CT spinule into an SBB. Red arrows point to clathrin coats. (C3) Enlargement of CT spinule shown in panel (C2) with red arrow pointing to clathrin coat. (D1,D2) Two consecutive images showing the lower end of a postsynaptic spine invaginating a CT spinule into an SBB. Red arrows point to clathrin coats. (D3) Enlargement of CT spinule shown in panel (D2), showing clathrin coat (red arrow) surrounding CT spinule from postsynaptic spine. Scale bars (A2,A3,B2,B3,C2,C3,D2) = 0.5 μm; Scale bar (D3) = 0.25 μm. [file Image_1.TIF]
